# Vitamins in Gynecologic Malignancies

**DOI:** 10.3390/nu16091392

**Published:** 2024-05-05

**Authors:** Natalia Wierzbowska, Tomasz Olszowski, Dariusz Chlubek, Mateusz Kozłowski, Aneta Cymbaluk-Płoska

**Affiliations:** 1 Department of Reconstructive Surgery and Gynecological Oncology, Pomeranian Medical University in Szczecin, Al. Powstańców Wielkopolskich 72, 70-111 Szczecin, Poland; 2 Department of Hygiene and Epidemiology, Pomeranian Medical University in Szczecin, Al. Powstańców Wielkopolskich 72, 70-111 Szczecin, Poland; 3 Department of Biochemistry and Medical Chemistry, Pomeranian Medical University in Szczecin, Al. Powstańców Wielkopolskich 72, 70-111 Szczecin, Poland

**Keywords:** retinoic acid, all-trans retinoic acid, 4-HPR, TAC-101, calcitriol, VDR, FR-α, FITC

## Abstract

The combination of vitamin A and D derivatives with classical chemotherapeutic treatments results in more satisfactory outcomes. The use of drug combinations, such as 9cUAB130 with carboplatin and cisplatin with TAC-101, shows enhanced cytotoxic effects and reductions in ovarian tumor volume compared to single-drug treatments. Combining cisplatin with calcitriol and progesterone increases VDR expression, potentially enhancing the effectiveness of anticancer therapy in ovarian cancer. The effectiveness of vitamin derivatives in anticancer treatment may vary depending on the characteristics of the tumor and the cell line from which it originated. An increase in thiamine intake of one unit is associated with an 18% decrease in HPV infection. Higher intake of vitamin C by 50 mg/day is linked to a lower risk of cervical neoplasia. Beta-carotene, vitamin C, and vitamin E are associated with risk reductions of 12%, 15%, and 9% in endometrial cancer, respectively. A balanced daily intake of vitamins is important, as both deficiency and excess can influence cancer development. It has been observed that there is a U-shaped relationship between group B vitamins and metabolic markers and clinical outcomes.

## 1. Introduction

Cancer is one of the leading causes of death among women in Europe. According to the findings of the European Cancer Information System, by 2040 the incidence of cancer among women will relatively increase by 14.79% compared to 2022 (Table 1) [1]. Among the most important reasons for this are demographic changes, such as an aging population. This increases the overall risk of cancer [1]. The incidence statistics may also be influenced by advances in diagnostics, allowing cancers to be detected at an earlier stage of development.

Strategies for preventing the development of gynecologic cancers include regular gynecologic check-ups, human papillomavirus (HPV) vaccination or physical activity and a healthy diet [2,3]. In the past two decades, many publications have focused attention on the role of micronutrients in the prevention of gynecologic malignancies [4,5,6]. Extensive meta-analyses have shown that daily supplementation with vitamin A and D3 can reduce the incidence of ovarian cancer [7,8,9], as well as mortality from malignant cancer by up to 12% [9].

Currently, only the vitamin A metabolite—all-trans-retinoic acid (ATRA)—appears on the list of drugs approved by the Food and Drug Administration (FDA) for the treatment of malignant neoplasm, specifically acute promyelocytic leukemia (APL) [10]. In the treatment of gynecologic malignancies, vitamins and their metabolites have only an adjunctive, but often extremely important function [11,12,13]. In this review, we will summarize the current state of scientific knowledge on the importance of vitamins in the development of the three most common gynecological malignancies (endometrial cancer, ovarian cancer and cervical cancer).

## 2. Methodology

To minimize the risk of bias, a thorough analysis of the available scientific literature was conducted, partly using PRISMA guidelines [14]. The criteria we used included: A database search (including PubMed and Google Scholar) covered the period from January 2000 to December 2023. Additionally, materials from the European Cancer Information System (https://ecis.jrc.ec.europa.eu/ (accessed on 4 January 2024)) were reviewed. The following keywords were applied: ‘vitamin’, ‘ovarian cancer’, ‘endometrial cancer’, ‘cervical cancer’, ‘vitamin A’, ‘vitamin D’, ‘vitamin E’, ‘vitamin K’, ‘folate’, and ‘B vitamins’. References of retrieved studies were searched manually for additional studies and reviews. No language restrictions were applied. Data were abstracted on prespecified forms. Only clinical studies that detailed the importance of vitamins in the development, prevention, and treatment of the three most common gynecological malignancies (endometrial cancer, ovarian cancer, and cervical cancer) and were available in full text, were deemed suitable for inclusion. The criteria for inclusion in the systematic review required clear reporting on the study’s design, characteristics of the participants, the regimen and duration of therapy. Data regarding these aspects of the included studies were meticulously extracted. Reviews and meta-analysis were also considered sources of citations of relevant studies and interpretation of their results. After a systematic search 1779 citations were identified from PubMed. Citations that were duplicates, multiple, or reviews lacking relevant information were excluded. Eventually, 108 papers discussing vitamins in the development, prevention, and treatment of the three most common gynecological malignancies (endometrial cancer, ovarian cancer, and cervical cancer) were considered eligible for inclusion in the systematic review.

## 3. Vitamin A

### 3.1. Mechanism of Action

**Figure 1:**
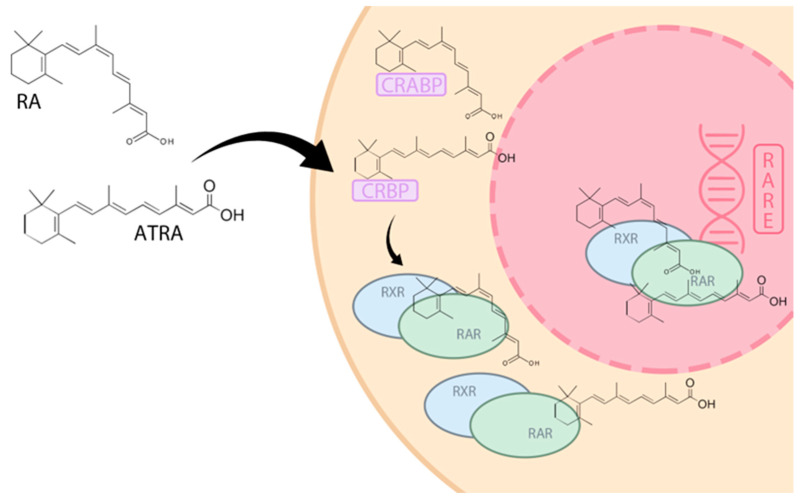
Signaling pathway of vitamin A metabolites. RA—retinoic acid, ATRA—all-trans retinoic acid, CRBP—cellular retinol binding proteins, CRABP—cellular retinoic acid binding proteins, RAR—retinoic acid receptor, RXR—retinoid X receptor, RARE—retinoic acid response element.

Vitamin A metabolites play a key role in the growth and differentiation of cells, including cancerous ones. Retinoic acid (RA) binds to the retinoic acid receptor (RAR) or retinoic X receptor (RXR) and penetrates into the cell nucleus [15]. Tumor Necrosis Factor alpha (TNF-α), TGF-β (Transforming Growth Factor beta), IFN-γ (Interferon gamma), and MAPK (Mitogen-Activated Protein Kinases) dependent pathways are some of the ways that control gene expression within the cell nucleus (Figure 1) [15]. It appears that intracellular bioavailability of retinoids is regulated by specific cellular retinol binding proteins (CRBPs) and cellular retinoic acid binding proteins (CRABPs). Doldo et al. reported reduced expression of CRBP-1 in grade 2 (moderately differentiated) and grade 3 (poorly differentiated) ovarian cancer [16]. Loss of CRBP-1 expression occurred regardless of stage, at every stage of ovarian cancer progression [16]. In addition, loss of CRBP-1 expression was associated with the malignant phenotype of tumor cells [16].

Retinoic acid is involved in the regulation of gene expression and signaling pathways in cells through its inhibitory effects against cyclin D1, human telomerase reverse transcriptase gene (hTERT), epidermal growth factor receptor (EGFR) or vascular endothelial growth factor (VEGF), among others [17]. Studies show that serum VEGF can be an important prognostic marker in ovarian cancer patients who have achieved complete clinical remission after chemotherapy [18]. There is ample evidence that retinoids, as part of cancer immunotherapy, have a role in reducing VEGF [19].

### 3.2. Ovarian Cancer

All-trans-retinoic acid (ATRA) affects aldehyde dehydrogenase 1 (ALDH1) activity, which is considered a key marker of malignant cancer stem cells, including ovarian cancer [11]. ATRA decreases signaling in the ALDH1/FoxM1/Notch1 pathway, thereby inhibiting tumor expansion in ovarian cancer. Interestingly, ATRA treatment reduces the proportion of ALDH1-positive cells. This contrasts with paclitaxel, which targets ALDH1-negative cell populations [11]. These findings suggest that the association of paclitaxel with ATRA may increase the success of anticancer therapy in ovarian cancer.

Retinoids increase the number of receptors for interleukin-2 (IL-2), which is responsible for mobilizing the immune system in the fight against cancer [20,21]. Retinoids, along with IL-2, increase the synthesis of IFN-γ, considered an important component of anti-tumor immune therapy due to its inhibition of angiogenesis, among other things [20,21]. Studies indicate the efficacy of the combination of IL-2 and 13-cis-retinoic acid as part of maintenance immunotherapy in patients who have achieved clinical benefit after treatment with either liposomal doxorubicin or oxaliplatin chemotherapy [22]. The use of the IL-2/RA drug combination resulted in a statistically significant improvement in progression-free survival (PFS) and overall survival (OS) curves [22].

The role of synthetic retinoid derivatives in the chemoprevention and treatment of ovarian cancer has been a subject of research for many years. In certain clinical cases, synthetic retinoid derivatives may have advantages over natural ones like ATRA. Fenretinide (R) and CD437 promote apoptosis of ovarian cancer cells [23,24]. Their action is to increase the activity of caspase-3 and caspase-9 enzymes in both ATRA-sensitive (CAOV-3) and resistant (SKOV-3) cells [23,24]. In addition, 4-HPR and CD437 increase the expression of proapoptotic genes and mitochondria uncoupling protein in OVCA433 cells [23,24]. It seems interesting that the use of 4-HPR in the preoperative period does not provide significant clinical benefit [25].

Depending on the cell line, synthetic retinoids appear to exhibit varying degrees of efficacy. Whitworth et al.’s investigation showed that combined therapy with carboplatin and the retinoid 9cUAB130 was superior to using each drug alone in the A2780 cell line [12]. Together, treatment with 9cUAB130 and carboplatin resulted in increased cytotoxicity against A2780 cells and a reduction in the expression of markers associated with cancer stem cells (CSCs) [12].

Clear cell adenocarcinoma exhibits a poor response to chemotherapy with cisplatin and paclitaxel, in contrast to other subtypes of epithelial ovarian cancer [26]. Research has demonstrated that in clear cell ovarian adenocarcinoma cell lines such as RMG-I and RMG-II, a synthetic retinoid called TAC-101 promotes apoptosis [26]. Cisplatin and TAC-101 have slightly different mechanisms of apoptosis induction [26]. Therefore, their simultaneous use may increase the success of anticancer therapy in clear cell ovarian cancer. A study in mice showed that the combination of these drugs resulted in a significant reduction in tumor volume [13]. These results have been confirmed for human ovarian cancer samples, both for cisplatin-sensitive tumors and RMG-I and RMG-II tumors [13].

### 3.3. Endometrial Cancer

Studies on endometrial cancer cell lines have shown that ATRA simultaneously affects RARα and RARβ [27]. Thus, it inhibits proliferation and induces apoptosis of RL95-2 cells [27]. It appears that this effect is mainly associated with RARβ expression, which was confirmed using a RARβ agonist (BMS453) [27]. This effect was not observed for HEC1A cells [27]. Studies show that RARα expression predominates in endometrioid adenocarcinoma cells [28]. However, with RA, increased expression of RARβ is possible [28].

According to a study by Mittal et al., fenretinide causes an increase in RA absorption, which leads to apoptosis in endometrial cancer cells by upregulating the expression of the STRA6 gene [29]. RA induces the expression of 17β-Hydroxysteroid Dehydrogenase Type 2 (HSD17B2). HSD17B2 is involved in the transition of the estrogen-dependent proliferative phase of the endometrium to the progesterone-dependent secretory phase [30]. Induction of HSD17B2 expression may find its application in therapy and chemoprevention of endometrial cancer.

### 3.4. Cervical Cancer

A 2012 meta-analysis found that an increased intake of vitamin A (particularly carotene and carotenoids) was associated with a reduced risk of cervical cancer [31]. Subsequent studies have not confirmed the efficacy of retinoids in the prevention of cervical intraepithelial neoplasia (CIN). Retinoids appeared to be ineffective in inducing regression of CIN3 [32]. At the same time, an inhibitory effect of retinoids on CIN progression was observed [32]. Sanusi’s study showed that the addition of vitamin A to neoadjuvant chemotherapy (NAC) improved the efficacy of the therapy, as evidenced by achieving a greater reduction in cervical cancer volume [33].

## 4. Vitamin D

### 4.1. Mechanism of Action

Vitamin D, or more specifically its biologically active form, calcitriol (1α,25(OH)₂D₃), has proven anticancer and anti-inflammatory effects [34,35]. 1α,25(OH)₂D₃ inhibits the growth of cancer cells, induces their apoptosis, and blocks the formation of new blood vessels [34,35]. As a result, the action of 1α,25(OH)₂D₃ limits tumor expansion. In addition, it promotes proper differentiation of newly formed cells and regulates the activity of the immune system [34,36]. 1α,25(OH)₂D₃, directly and indirectly (by affecting pro-inflammatory prostaglandins) inhibits aromatase expression. Such effects may provide the basis for its potential use in the therapy and chemoprevention of estrogen-dependent cancers [34].

**Figure 2:**
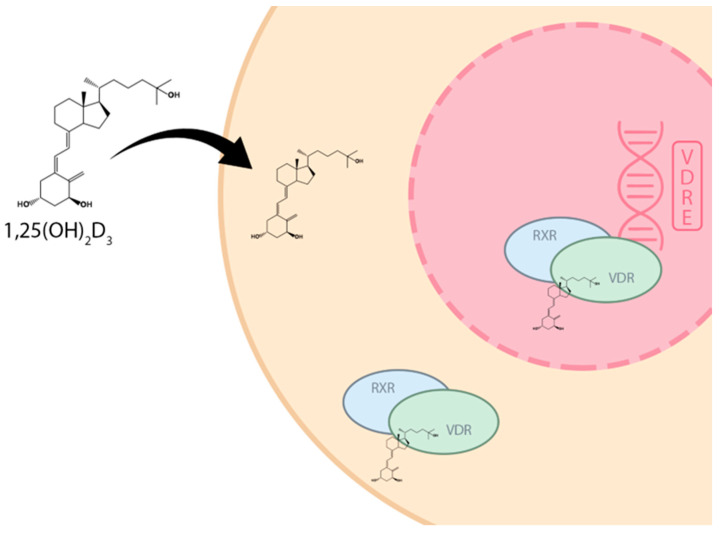
Signaling pathway of the active form of vitamin D. 1α,25(OH)₂D₃—calcitriol, VDR—vitamin D receptor, RXR—retinoid-X-receptor, VDRE—vitamin D response element.

The human body’s numerous tissues contain vitamin D receptors (VDRs). Dendritic cells, macrophages, and T and B lymphocytes have the VDR localized on their surface, indicating that vitamin D plays a role in the immunological response [37]. Myometrial and endometrial cells also express VDR [38]. Following its binding to VDR, 1α,25(OH)₂D₃ translocates as a VDR-RXR complex into the cell nucleus. The vitamin D response elements (VDREs) in the cell nucleus are bound by the VDR complex with RXR, which influences gene transcription (Figure 2) [35]. It has been suggested in recent years that the phase of the menstrual cycle may affect the expression of VDR in endometrial cells. However, the findings are inconclusive. Some studies indicate that VDR expression may be lower during the proliferative phase than in the secretory phase [39], while others suggest that VDR expression is lower in the midway secretory phase compared to the early secretory phase [40].

### 4.2. Endometrial Cancer

Overexpression of the CYP24A1 oncogene in cancer cells is considered a poor prognostic factor [41,42]. CYP24A1 protein through degradation of 1α,25(OH)₂D₃ inhibits anti-tumorigenic effects. A study by Bokhari et al. showed that progestogens, commonly used in the endocrine treatment of endometrial cancer, are inhibitors of CYP24A1 [41]. Thus, the association of progesterone with 1α,25(OH)₂D₃ allows for enhanced anti-tumorigenic effects [41].

A comprehensive meta-analysis showed that hypovitaminosis D is common among patients with gynecologic malignancies (including endometrial cancer) [43]. Such findings suggest that vitamin D supplementation may be part of the prevention of endometrial cancer. However, it should be noted that vitamin D deficiency is favored by obesity, and this is one of the main risk factors for endometrial cancer [44,45]. To reach the same serum 25-hydroxyvitamin D levels as adults of normal weight, obese individuals require larger loading doses of vitamin D [46].

### 4.3. Ovarian Cancer

Overexpression of 24-hydroxylase mRNA has also been reported in breast and ovarian cancers [41]. Intriguing findings in this context have emerged from animal models. It has been observed that 1α,25(OH)₂D₃ therapy induces CYP24A1 expression in the ovaries of mice [47]. However, when 1α,25(OH)₂D₃ is combined with progesterone, there is a decrease in CYP24A1 expression [47]. The presence of progesterone receptors on the cell surface was essential for this combination of medications to be effective. In the absence of these receptors, no effect was observed.

Research conducted on animal models has shown that the active metabolite of vitamin D, 1α,25(OH)₂D₃, plays a role in regulating ovarian function [48]. Similar findings have been observed in studies using human oocytes [49]. It has been demonstrated that 1α,25(OH)₂D₃ can inhibit the unchecked proliferation of ovarian cancer cells at the G1/S and G2/M checkpoints of the cell cycle [50,51]. This inhibitory effect is attributed to its influence on GADD45 (growth arrest and DNA damage-inducible 45) and the cyclin-dependent kinase inhibitors P21 and P27 [50,51].

It has been discovered that 1α,25(OH)₂D₃ suppresses the migration and invasion of SKOV-3 cells and encourages the cells to take on an epithelial phenotype when treated with TGF-β1 [52]. Migration is inhibited by 1α,25(OH)₂D₃ via lowering the expression of epithelial mesenchymal transition (EMT) markers [52]. Elevated VDR expression in ovarian cancer cells is linked to calcitriol-induced increases in E-cadherin and decreases in vimentin levels [52].

A significant finding concerns the effect of a variation in the VDR gene on the risk of developing ovarian cancer. The FokI polymorphism has been linked to an increased risk of ovarian cancer compared to four other polymorphisms (ApaI, BsmI, Cdx-2, and TaqI), as revealed by a comprehensive meta-analysis [53]. This polymorphism results in two distinct forms of the VDR protein: a longer version (f-VDR) and a shorter version (F-VDR) [54]. The f variant leads to reduced sensitivity of the VDR protein to 1α,25(OH)₂D₃, diminishing its NF-kB transcriptional activity [54]. This reduction in activity is associated with a weaker immune response and decreased expression of IL-12 [54].

An in vitro study on ovarian cancer cells revealed that a combination of progesterone, calcitriol, and cisplatin significantly enhanced the effectiveness of anticancer therapy [55]. The triple-drug combination was observed to promote apoptosis by downregulating the expression of BCL2 and PARP-1, upregulating caspase-3 and BAX, and inhibiting the PI3K/AKT and MAPK/ERK signaling pathways [55]. Additionally, a reduction in the expression of SMAD2/3, ABC transporters (ABCG1 and ABCG2), and the multidrug resistance protein-1 (MDR-1) was associated with increased responsiveness to treatment [55]. Paucarmayta et al. have suggested using TGF-β and CYP24A1 signaling proteins to monitor the effectiveness of combination therapy with progesterone and calcitriol [55]. Conversely, further research indicates that higher expression of VDR is associated with lower overall survival rates in ovarian cancer patients [56]. Additionally, it has been proposed that cytoplasmic VDR expression may serve as an independent prognostic factor [56].

### 4.4. Cervical Cancer

Cervical cancer cells show a higher expression of VDR than cells of healthy tissue, but it is not recommended as a prognostic factor for cervical cancer [57]. Depending on the cell line derived from cervical cancer, differences in VDR activity are observed. C33A cells show resistance to the effect of calcitriol on VDR expression [58]. Recent studies have shown that certain VDR polymorphisms are associated with a high risk of HPV16-associated CIN2 (cervical intraepithelial neoplasia grade 2) and cervical cancer [59]. Among them are Fok1 and Taql, whose expression also increases the risk of ovarian malignancies [60]. A study by Vahedpoor et al. showed that in patients with CIN1, taking one dose of 50,000 IU of vitamin D every 2 weeks for 6 months has a supportive effect on regression of dysplastic lesions [61]. In contrast, Punchoo et al. noted that already physiological doses of 25-hydroxyvitamin D are sufficient to inhibit proliferation and to stimulate apoptosis in cells from the SiHa lineage [62].

## 5. B Vitamins

Acting as coenzymes, folic acid and vitamin B12 are involved in numerous biological processes. They play critical roles in the synthesis of purines and pyrimidines needed for DNA synthesis and in the metabolism of homocysteine [63]. The impact of folate on the risk of developing cancer in women remains a controversial topic. To date, most of the research [64,65,66] has not specifically examined the impact of dietary folate on the risk of endometrial and ovarian cancer. The results from studies on folate supplementation have been mixed. Vitamins B2, B6, and B12, along with supplemental folate, appeared to increase the incidence of type II endometrial cancer [67]. B vitamins are involved in single-carbon metabolic pathways that are crucial for methylation and DNA synthesis [63]. When administered in excess, they can be used for growth by rapidly dividing cancer cells and increase their proliferation [68]. On the other hand, B vitamin deficiency can also contribute to cancer development. B vitamin deficiency can affect DNA instability resulting from DNA hypomethylation, disruption of DNA precursors, inappropriate incorporation of uracil into DNA and chromosome breaks [69]. Based on the studies, the association of B vitamin with metabolic markers and U-shaped clinical outcomes was observed [70].

Insightful findings were derived from an analysis by Wien et al. of randomized controlled trials (RCTs) [71]. This analysis suggested that a daily folate dosage range of 0.4–1 mg is associated with a higher risk of cancer compared to supplementation exceeding 1 mg/day [72]. More recent studies have indicated that women with endometriosis who consume higher amounts of folate are at an increased risk of developing ovarian cancer [72]. Such results were not observed in women without endometriosis [72]. Additionally, a worse prognosis in cervical cancer is associated with elevated expression of the folic acid receptor alpha (FRα) [73]. Folate FITC (5-fluorescein isothiocyanate) appears to be utilized in intraoperative fluorescence imaging [74]. This agent, when administered systemically, targets the FR-α receptor enabling precise real-time detection of tumor tissue during surgery in patients with FR-positive ovarian tumors [74].

A detailed analysis of the data indicated that a high dietary intake of folate may be associated with a reduced risk of ovarian and endometrial cancer [75]. Similarly, a high consumption of vitamin B6 from the diet may be associated with a decreased risk of ovarian cancer [75]. Furthermore, a study utilizing the National Health and Nutrition Examination Survey (NHANES) database suggests that consuming 2 mg of thiamine (vitamin B1) daily may help in preventing HPV infection [76]. According to a study by Piyathilake et al., higher plasma concentrations of folate and vitamin B12 in women infected with HPV are associated with a reduced risk of developing CIN2+ [77]. This effect is thought to be due to persistent elevated methylation of the HPV 16 E6 promoter at CpG sites [77]. A study published in 2024 confirmed the association between methylation levels and the risk of CIN3+ [78].

Findings on the role of folate and vitamin B12 in the development of HPV infection remain inconsistent. Many confirm a negative association between blood levels of the vitamins and the risk of developing hrHPV infection [79,80,81], while others find no such relationship [82,83].

## 6. Vitamin C, E and K

Vitamin C and E are antioxidants that reduce the levels of reactive oxygen species (ROS) and prevent their excessive accumulation within tissues [84]. ROS are responsible for DNA damage, leading to genomic instability. Studies show that cancer cells produce increased amounts of ROS [84]. This stimulates their growth and proliferation, promotes the formation of new blood vessels, and increases the risk of resistance to chemotherapy and cancer recurrence [84]. Research indicates that consuming a diet rich in antioxidants, such as vitamins A, C, and E, may reduce the risk of developing cancer in women [85]. A comprehensive meta-analysis has linked increased intake of beta-carotene, vitamin C, and vitamin E to a significantly lower risk of developing endometrial cancer [85]. However, a large cohort study published a few years later challenged the assertion that these vitamins contribute to a lower incidence of endometrial cancer [86]. Moreover, more recent studies have refuted the notion that intake of vitamins A, C, or E prior to an ovarian cancer diagnosis is associated with improved survival and a decreased risk of the disease [87,88,89].

The theory proposed by Barchitta et al. suggests that women who report an increased intake of dietary antioxidants are associated with a lower incidence of hrHPV (high-risk human papillomavirus) infection [90]. However, further research is needed as the study’s conclusions are not substantiated by any data. Supporting this hypothesis, the findings by Zheng et al. provide some evidence of a negative correlation between vitamin C consumption and HPV infection [91]. A negative correlation between certain factors and cervical cancer was observed only in women aged 25 years and older, with no similar correlation found in women under 25 [91]. An analysis in 2016, which reviewed 12 randomized trials including 1 cohort study and 11 case-control studies, identified a negative association between vitamin C intake and the incidence of cervical cancer [92]. Cao et al. reported that even a daily intake of as little as 50 mg of vitamin C is associated with a significant reduction in the risk of cervical cancer [92].

The first direct evidence linking ascorbate to the metabolism of human cancer cells was provided by Kuiper et al. [93]. In 2010, researchers found a compelling result: there was a strong negative correlation between ascorbate levels and the activation markers of hypoxia-inducible factor-1 (HIF-1), including VEGF, HIF-1α, GLUT-1, and BNIP3 [93]. The results suggest that the deficiency of ascorbate as a source of hydroxyl groups in tissues, hinders the control of the HIF-1 pathway [93]. This situation favors the progression of endometrial cancer and the promotion of its aggressive phenotypes. Among the mechanisms of ascorbate’s prooxidant action within ovarian cancer cells, Ma et al. singled out participation in hydrogen peroxide synthesis, activation of the ATM/AMPK pathway or inhibition of the mTOR pathway [94].

The carcinoprotective role of antioxidants is still a topic of research. One study published several years ago confirmed the association between high vitamin E intake and reduced risk of cervical cancer [95,96]. Of the vitamin E isoforms, the tocotrienol isoforms (α, β, γ and δ) exhibit particular anticancer properties [97]. The mechanisms by which vitamin E operates include the inhibition of the NF-κB pathway, suppression of 3-hydroxy-3-methylglutaryl-coenzyme A (HMG-CoA) reductase activity, and neutralization of reactive nitrogen and oxygen species [97]. Despite these actions, a review of randomized controlled clinical trials from 2012 to 2022 did not conclusively confirm the beneficial effects of vitamin E in treating cancers, particularly those affecting the female genital tract [98]. While vitamin E has been proposed as a potential cancer preventative agent, there is evidence suggesting that antioxidants might, in some cases, enhance the survival and proliferation of cancer cells [99,100].

There are not many studies in the scientific literature discussing the effects of vitamin K on cancer cells. Wang et al. demonstrated that the risk of developing CIN2+ is associated with low vitamin K intake [101]. Studies have shown that vitamin K, by increasing ROS levels, can promote apoptosis of SiHa (HPV 16-transformed cervical cells) or SKOV-3 (ovarian cancer cell line) cancer cells [102]. However, a study by Zhu et al. found no association of vitamin K with the development of gynecologic cancers [103]. Although there is growing interest in the role of vitamins in cancer prevention and treatment, specific data on the role of vitamin K remains inconsistent and very limited compared to other vitamins.

## 7. Potential Risks and Side Effects

Excessive vitamin supplementation can lead to hypervitaminosis. This is especially true in the context of fat-soluble vitamins (such as A, D, E, K), excess of which is stored in tissues [104]. The most commonly reported cases of hypervitaminosis involve vitamins A and D [104]. When considering vitamin supplementation, it is important to note the potential risks and side effects associated with high doses of vitamins (Table 2). 

## 8. Limitations

This study includes several important limitations that may affect the interpretation of the results from the literature review on vitamins in gynecological malignancies. First and foremost, the heterogeneity of vitamin measurement methods used in various studies introduces considerable variability, which can lead to biased results and comparisons. The diversity of measurement methodologies and reporting standards across studies makes it difficult to draw uniform conclusions.

Additionally, our review was not exhaustive due to the selective criteria used to choose the included studies. This selection, based on the significance and impact of the studies, may lead to the omission of some lesser-known but potentially relevant works. Such a limitation affects the comprehensiveness of our analysis and may distort the interpretation of the overall effectiveness and reliability of vitamin research.

It is also worth noting that we applied only part of the PRISMA guidelines in our work. This means we did not follow all the recommendations for systematic reviews, which affects the methodological rigor of our work. Consequently, our study should be considered a literature review rather than a full-fledged systematic review. The scope of this review did not allow for a meta-analytic approach, which limits our ability to quantitatively synthesize data from multiple studies.

Using standardized criteria for selecting and comparing methods used to measure the investigated vitamins could also help reduce heterogeneity and improve the comparability of results between different studies.

## 9. Summary and Conclusions

Studies have shown that there is a synergistic effect between vitamin A derivatives and chemotherapeutic agents in promoting apoptosis of cancer cells and inhibiting their proliferation (Table 3). The effectiveness of synthetic retinoids may vary from one cell line to another. Vitamin A metabolites have a downward effect on the expression of CSCs and prognostic markers such as VEGF, ALDH-1.

The response to treatment with an active form of vitamin D depends on VDR activity. VDR activity differs within healthy and cancerous tissue, and between cancer cell lines. Studies have shown that by combining cisplatin with calcitriol and progesterone, VDR expression can be increased (Table 3). This has implications for the effectiveness of anticancer therapy in ovarian cancer.

It has been observed that there is a U-shaped relationship between group B vitamins and metabolic markers and clinical outcomes. B vitamins both in excess and in deficiency can promote the development of cancers, including gynecological cancers. A deficiency of B vitamins can cause DNA instability, while an excess can be used for the proliferation of rapidly dividing cancer cells. Therefore, it is important to achieve an adequate balance of daily intake of B vitamins.

Vitamins C, E and K show beneficial anti-cancer properties due to their involvement in neutralizing and fighting free radicals. Nonetheless, studies from previous years have yielded conflicting results regarding the effects of vitamins with antioxidant properties on the development of female-specific cancers. This indicates the need for further research in this area.

In conclusion, to date there is no official medical indication for the use of vitamins as a direct treatment for gynecological cancers. There is scientific evidence for the effectiveness of vitamins in preventing the development of gynecologic cancers, although it is still not consistent (Table 4). Nevertheless, in the context of preventing various cancers, maintaining a healthy diet rich in fruits, vegetables, and whole grains is fundamental because it provides many of the vitamins needed to support overall health.

## Figures and Tables

**Table 1:** Incidence and mortality from the three most common gynecologic cancers [1].

Cancer	Estimates of Cancer Incidence in 2022 in Europe	Estimates of Cancer Mortality in 2022 in Europe	Estimated Relative Change of Incidence from 2022 to 2040	Estimated Relative Change of Mortality from 2022 to 2040
Corpus uteri	5.40%	3.00%	13.00%	24.80%
Ovary	3.20%	4.80%	12.90%	19.20%
Cervix uteri	-	-	2.60%	12.00%

**Table 2:** Potential risks and side effects of hypervitaminosis [105,106,107,108].

Vitamin	Potential Risks and Side Effects
Vitamin A	An acute form: headache, visual disturbances, vomiting, short-term loss of consciousness, dizziness, irritability, gastrointestinal disturbance, fever, skin rashes Chronic forms: hepatotoxicity and teratogenicity, kidney failure, arrhythmia, arthralgia, deterioration of vision, lipid disorders, dermatological disorders (including carotenodermia)
Vitamin D	greater risk of pancreatic cancer, cardiovascular events, tremors, muscle pain, subcutaneous hemorrhages, dehydration, dental enamel hypoplasia
Vitamin E	bleeding, hemorrhages, aggravation of angina, hypertension, atherosclerosis, gastrointestinal disturbance, fatigue, weakness, headache, dysregulation of immune system, delayed wound healing
Vitamin K	reduction in blood sugar levels, changes in blood clotting times, hemolytic anemia, jaundice, liver damage
Vitamin C	kidney disease, stomach ulcer, disturbance of the pancreas, gallstones, kidney stones, gastrointestinal disturbances (diarrhea, nausea, vomiting, heartburn, stomachache), increased blood pressure, hormonal disorders, leukopenia, insomnia, headache, deterioration of vision, feelings of weakness, dizziness, allergic reactions to skin
Vitamin B1 (thiamine)	interference in the thyroid function, heart failure, paralysis, restlessness, convulsions
Vitamin B2 (riboflavin)	hepatotoxicity, cardiomyopathies, vomiting, hypotension, fatigue, photophobia, paresthesia, itching, cracks and ulcers in the corners of the mouth
Vitamin B3 (niacin)	hepatotoxicity, aggravation of bronchial asthma, hypotension, dizziness, gout, fasting hyperglycemia, gastrointestinal disturbances, dermatological disorders, insomnia
Vitamin B6 (pyridoxine)	neurological symptoms, gastrointestinal disturbances, photosensitivity, skin rashes
Vitamin B9 (folic acid)	increased risk of diabetes (in interaction with lowest levels of vitamin B12), gastrointestinal disturbances, sleep disorders, dermatological disorders
Vitamin B12 (cobalamin)	cardiovascular disorders: congestive heart failure, pulmonary edema, palpitations, allergic reactions, paresthesia

**Table 3:** The role of selected vitamins and their metabolites in the treatment of gynecologic cancers.

Reference	Vitamin/Vitamin Metabolite	Role
OVARIAN CANCER		
Young et al. [11]	ATRA	ATRA decreases signaling in the ALDH1/FoxM1/Notch1 pathway. ATRA reduces the proportion of ALDH1-positive cells, unlike paclitaxel, which targets the ALDH1-negative cell population.
Whitworth et al. [12]	9cUAB130	Combined treatment with 9cUAB130 and carboplatin achieves greater cytotoxicity against A2780 cells, as well as a decrease in the expression of CSCs markers.
Ezawa et al. [13], Suzuki et al. [26]	TAC-101	The combination of cisplatin and TAC-101 allows a significant reduction in the volume of clear cell ovarian cancer, both in cisplatin-sensitive tumors and in RMG-I and RMG-II tumors.
Prabhala et al. [20], Bono et al. [21]	RA	Retinoids, together with IL-2, increase the synthesis of the anti-tumor IFN-γ.
Recchia et al. [22]	RA	The combination of IL-2 and 13-cis-retinoic acid has shown efficacy as maintenance immunotherapy in patients who have achieved clinical benefit after treatment with either liposomal doxorubicin or oxaliplatin chemotherapy.
Brewer et al. [23], Holmes et al. [24]	4-HPR, CD437	4-HPR and CD437 promote apoptosis of ovarian cancer cells by increasing the activity of caspase-3 and caspase-9 enzymes in both ATRA-sensitive (CAOV-3) and resistant (SKOV-3) cells, as well as increasing the expression of proapoptotic genes and mitochondria uncoupling protein in OVCA433 cells.
Colombo et al. [25]	4-HPR	The use of 4-HPR in the preoperative period does not provide significant clinical benefit.
Paucarmayta et al. [55]	1α,25(OH)₂D₃	The association of progesterone and calcitriol with cisplatin increases the efficacy of anticancer therapy.
Chen et al. [50], Jiang et al. [51]	1α,25(OH)₂D₃	1α,25(OH)₂D₃ has the ability to stop uncontrolled growth of ovarian cancer cells at the G1/S and G2/M checkpoint of the cell cycle.
Hou et al. [52]	1α,25(OH)₂D₃	When SKOV-3 cells are stimulated with TGF-β1, 1α,25(OH)₂D₃ effectively suppresses their migration and invasion, while also promoting the adoption of an epithelial phenotype. This is achieved by 1α,25(OH)₂D₃ through its inhibition of cell migration, which it accomplishes by reducing the expression of EMT factors.
Kuznia et al. [9]	1α,25(OH)₂D₃	Vitamin D administered daily reduced cancer mortality by 12 %.
ENDOMETRIAL CANCER		
Tsuji et al. [27]	ATRA	ATRA inhibits proliferation and induces apoptosis of RL95-2 cells while affecting either RARα or RARβ, with the effect mainly on RARβ expression.
Ito et al. [28]	RA	RA allows increased expression of RARβ relative to RARα in endometrioid adenocarcinoma cells.
Mittal et al. [29]	4-HPR	4-HPR by increasing STRA6 gene expression allows RA to increase uptake, which induces apoptosis of endometrial cancer cells.
Cheng et al. [30]	RA	The expression of HSD17B2, an enzyme that plays a crucial role in the metabolic conversion of hormones critical for the transition of the endometrium from the progesterone-dependent secretory phase to the estrogen-dependent proliferative phase, is induced by retinoic acid.
CERVICAL CANCER		
Sanusi et al. [33]	Vitamin A	Adding vitamin A to NAC achieves greater reduction in cervical cancer volume.
Vahedpoor et al. [61]	Vitamin D	Administering one dose of 50,000 IU of vitamin D every two weeks for a period of six months was found to have a supportive effect on the regression of dysplastic lesions in individuals diagnosed with CIN1.
Punchoo et al. [62]	25(OH)D₃	Already physiological doses of 25-hydroxyvitamin D are sufficient to inhibit proliferation and to stimulate apoptosis in cells of the SiHa lineage.

**Table 4:** The role of vitamins and their metabolites in the prevention of gynecologic cancers.

Reference	Article Type	Vitamin/Vitamin Metabolite and Daily Dose (If Reported)	Cancer Risk	Measure of Association
OVARIAN CANCER				
Wang et al. [7]	Meta-analysis	Vitamin A	Intake of vitamin A was inversely associated with risk of ovarian cancer, especially among North Americans.	RR = 0.816 (95% CI 0.723–0.920)
Liao et al. [8]	Meta-analysis	Vitamin D	Intake of vitamin D was inversely associated with risk of ovarian cancer.	RR = 0.80 (95% CI 0.67–0.95)
Gersekowski et al. [72]	Case-control study	Folate: 400+ μg	Higher dietary folate intake was associated with an increased risk of ovarian cancer for women with endometriosis. No association for women without endometriosis.	OR = 1.37 (95% CI 1.01–1.86)
Arthur et al. [75]	Case-control study	Folate: >560.7 μg Vitamin B6: >2.9 mg	Higher dietary folate intake was inversely associated with risk of ovarian cancer. Higher dietary intake of vitamin B6 was inversely associated with ovarian cancer risk.	Folate: HR_q4 vs. q1_ = 0.39 (95% CI: 0.19–0.80) Vitamin B6: HR_q4 vs. q1_ = 0.49 (95% CI: 0.24–0.98
ENDOMETRIAL CANCER				
Arthur et al. [75]	Case-control study	Folate: >614.9 μg	Higher dietary folate intake was inversely associated with risk of endometrial cancer.	HR_q4 vs. q1_ = 0.52 (95% CI 0.29–0.93)
Bandera et al. [85]	Meta-analysis	Beta-carotene, Vitamin C Vitamin E	Beta-carotene is associated with a 12% risk reduction in endometrial cancer, vitamin C is associated with a 15% risk reduction, and vitamin E is associated with a 9% risk reduction.	Beta-carotene: OR = 0.88 (95% CI: 0.79–0.98), Vitamin C: OR = 0.85 (95% CI: 0.73–0.98), Vitmain E: OR = 0.91 (95% CI: 0.84–0.99)
Zhu et al. [103]		Vitamin B12: 3.17 (1.78–5.14) Data were presented as median with range.	Intake of vitamin B12 was inversely associated with risk of endometrial cancer.	OR = 0.812 (95% CI: 0.714, 0.925)
CERVICAL CANCER				
Zhou et al. [76]	Secondary data analysis	Thiamine: 2 mg	An increase of every 1-unit rise in thiamine intake is associated with a 18% decrease in HPV infection.	β = 0.82 (95% CI: 0.78–0.86)
Barchitta et al. [90]	Cross-Sectional Study	Vitamin A: 1097.59 IU (538.14), Vitamin C: 116.71 mg (107.55), Vitamin E: 37.97 mg (23.44) Data were presented as median with interquartile range.	Higher dietary intake of vitamin A, C and E intake was inversely associated with risk of hrHPV infection.	Composite Dietary Antioxidant Index (CDAI): OR = 0.39 (95% CI: 0.18–0.85)
Zheng et al. [91]	Cross-Sectional Study	Vitamin C	Negative association between vitamin C intake and HPV infection in women 25 years of age and older.	OR = 0.7 (95% CI: 0.52–0.94)
Cao et al. [92]	Meta-analysis	Vitamin C: 50 mg	Increased vitamin C intake by 50 mg/day was related to the reduced risk of cervical neoplasia.	OR = 0.92 (95% CI: 0.89–0.94)
Hu et al. [95]	Meta-analysis	Vitamin E	Intake of vitamin E was inversely associated with risk of cervical neoplasia.	OR = 0.58 (95% CI: 0.47–0.72
Myung et al. [96]	Meta-analysis	Vitamin B12, Vitamin C, Vitamin E, Beta-carotene	Intake of vitamin E was inversely associated with risk of cervical neoplasia.	Vitamin B12: OR = 0.35 (95% CI: 0.19–0.63), Vitamin C: OR = 0.67 (95% CI: 0.55–0.82), Vitamin E: OR = 0.56 (95% CI: 0.35–0.88), Beta-karoten: OR = 0.68 (95% CI: 0.55–0.84)
Wang et al. [101]	Cohort study	Folate: 358.9 μg (283.8–836.5), Vitamin B6: 1.9 mg (1.6–4.2), Vitamin C: 59.4 mg (43.2–148.2), Niacin: 187.2 mg (127.7–560.6), Vitamin K: 187.2 μg (127.7–560.6) Data were presented as median with range.	The risk of CIN2+ was associated with low dietary intake of folate, vitamins B6, C, niacin, and vitamin K.	Folate: OR = 1.55 (95% CI: 1.03–2.33); Vitamin B6: OR = 1.63 (95% CI: 1.08–2.46), Vitamin C: OR = 1.59 (95% CI: 1.05–2.42), Vitamin B3: OR = 1.65 (95% CI: 1.08–2.51), Vitamin K: OR = 1.60 (95% CI: 1.05–2.44)
